# Biological Evaluation and Transcriptomic Analysis of Corylin as an Inhibitor of Osteoclast Differentiation

**DOI:** 10.3390/ijms22073540

**Published:** 2021-03-29

**Authors:** Anna Xiao-Dan Yu, Jian Xiao, Shi-Zheng Zhao, Xiang-Peng Kong, Kenneth Kin-Leung Kwan, Brody Zhong-Yu Zheng, Kevin Qi-Yun Wu, Tina Ting-Xia Dong, Karl Wah-Keung Tsim

**Affiliations:** 1 Shenzhen Key Laboratory of Edible and Medicinal Bioresources, HKUST Shenzhen Research Institute, Nanshan, Shenzhen 518000, China; xyuav@connect.ust.hk (A.X.-D.Y.); boxj@ust.hk (J.X.); xpkong@ust.hk (X.-P.K.); klkwan@connect.ust.hk (K.K.-L.K.); brodyz@ust.hk (B.Z.-Y.Z.); qwuah@connect.ust.hk (K.Q.-Y.W.); botina@ust.hk (T.T.-X.D.); 2 Division of Life Science and Center for Chinese Medicine, The Hong Kong University of Science and Technology, Clear Water Bay Road, Hong Kong 999077, China; szhaoaf@connect.ust.hk

**Keywords:** corylin, osteoclast, RANKL, transcriptomics, signaling pathway

## Abstract

Corylin, a flavonoid isolated from the fruit of *Psoralea corylifolia*, has an osteogenic effect on osteoblasts in vitro and bone micromass ex vivo. However, the effect and mechanism of corylin in regulating osteoclastogenesis remain unknown. By using murine bone marrow macrophages as the osteoclast precursor, corylin was found to inhibit the receptor activator of nuclear factor (NF) κB ligand (RANKL)-induced osteoclast differentiation via down-regulating osteoclastic marker genes. In parallel, F-actin formation and osteoclast migration were diminished in corylin-treated cultured osteoclasts, and subsequently the expressions of osteoclastic proteins were suppressed: the suppression of protein expression was further illustrated by transcriptomic analysis. Furthermore, corylin inhibited the nuclear translocation of p65, giving rise to a restraint in osteoclastic differentiation through the attenuation of transcription factors nuclear factor kappa-light-chain-enhancer of activated B cells (NF-κB) and nuclear factor of activated T cells c1 (NFATc1). There was no obvious change in apoptosis when the RANKL-induce osteoclasts were cultured in the presence of corylin. The finding supports the potential development of corylin as an osteoclast inhibitor against osteoporosis.

## 1. Introduction

Characterized by low bone mass density and degradative microarchitecture, osteoporosis is a systemic skeletal disease and age-related metabolic disorder. This medical problem is commonly occurring today, particularly in post-menopausal women and the aged population [1]. Considered as a “silent killer” disease, osteoporosis has been widely regarded as a major public health problem [2]. The so called “brittle bone” or “porous bone” increases the susceptibility to bone fracture that causes disability and chronic pain. The current treatments of osteoporosis, e.g., estrogen, bisphosphonates, and Parathyroid hormone (PTH), have limitations, including side effects, high cost, and poor patient compliance. Therefore, there is an urgent need to explore and identify better and safer drugs with lower costs [3].

Normal bone health is maintained in a balance between bone resorption by osteoclasts and bone formation by osteoblasts. Excessive activation of bone-resorbing osteoclasts is the dominant pathophysiology of post-menopausal and aging osteoporosis, resulting in greater bone resorption and thereafter less bone formation [4,5]. Therefore, an agent inhibiting bone resorption, as well as promoting bone formation simultaneously, should be a desirable strategy in therapy of osteoporosis. Osteoclasts, derived from hematopoietic cells of the monocyte/macrophage lineage stemming from bone marrow, can be identified according to their distinct morphological and phenotypic characteristics, i.e., multi-nucleated giant cell, ruffled border, and expression of tartrate-resistant acid phosphatase (TRAP) [6,7]. Interestingly, lysosomal enzymes, such as TRAP and cathepsin K (CTSK), are actively expressed by osteoclasts and then released into the bone-resorbing compartment to degrade the bone matrix [8]. Hence, TRAP and cathepsin K have been widely used as crucial markers of mature osteoclast, as well as in estimating the osteoclastic activity.

Two cytokines, namely macrophage colony-stimulating factor (MCSF) and receptor activator of nuclear factor κB ligand (RANKL), are indispensable for the survival and differentiation of osteoclast precursors, both in vitro and in vivo [9]. Notably, the RANKL signal in osteoclast precursors provokes the activation of mitogen-activated protein kinase (MAPK) and nuclear factor kappa-light-chain-enhancer of activated B cells (NF-κB) signaling pathways, followed by the activation of a cascade of transcription factors, including NF-κB, activator protein 1 (AP-1), and nuclear factor of activated T cells c1 (NFATc1), which result in the expression of genes corresponding to maturation of osteoclasts [10].

Corylin, a phytochemical deriving from the fruit of *Psoralea corylifolia*, has been demonstrated to be an effective component in exhibiting osteogenic effects in cultured osteoblasts in vitro and bone micromass ex vivo, and the corylin-induced osteoblastic differentiation was shown to be triggered via estrogen and Wnt/β-catenin signaling pathways [11]. Recently, it was reported that corylin inhibits osteoclastogenesis using RAW 264.7 cells and attenuated post-menopausal osteoporosis in mice [12]. However, the effects of corylin in regulating intracellular signaling pathways during osteoclastogenesis remain unrevealed. Corylin was shown to have anti-inflammatory properties, partially by suppressing the activation of NF-κB in LPS-induced inflammation and sepsis [13], and which attenuated the phosphorylation of MAPK in LPS-activated RAW 264.7 cells and BV2 cells [13,14]. On the other hand, the application of corylin in cultured osteoblasts could suppress the expression of RANKL [11]. Therefore, we hypothesize that corylin could inhibit RANKL-induced osteoclastogenesis via the suppression of NF-κB signaling. By using murine bone marrow macrophages as the osteoclast precursor, the mechanism of corylin on RANKL-induced osteoclastogenesis was examined in vitro by using biochemical assays and transcriptomics. This study provides experimental evidence that corylin exhibits potential as a novel natural product for the treatment of osteoclast-dominated diseases like osteoporosis.

## 2. Results

### 2.1. Corylin Inhibits the Differentiation of Bone Marrow Macrophages into Osteoclasts

**Figure 1:**
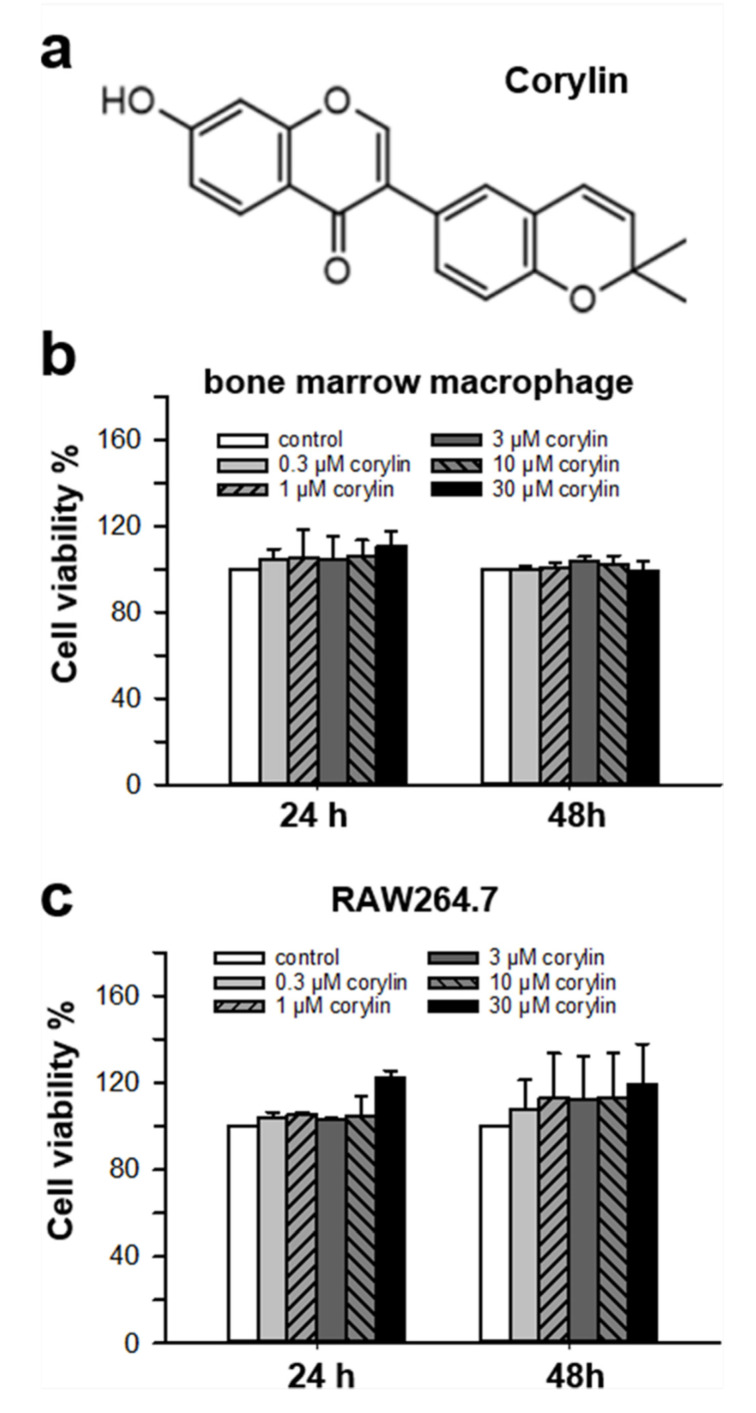
(**a**) Chemical structure of corylin. Cell viability of (**b**) cultured bone marrow macrophages or (**c**) RAW 264.7 cells treated with corylin for 24 h and 48 h, respectively. Data represent the mean ± SD, *n* = 4, each with triplicate samples. *p* < 0.05, compared with control.

**Figure 2:**
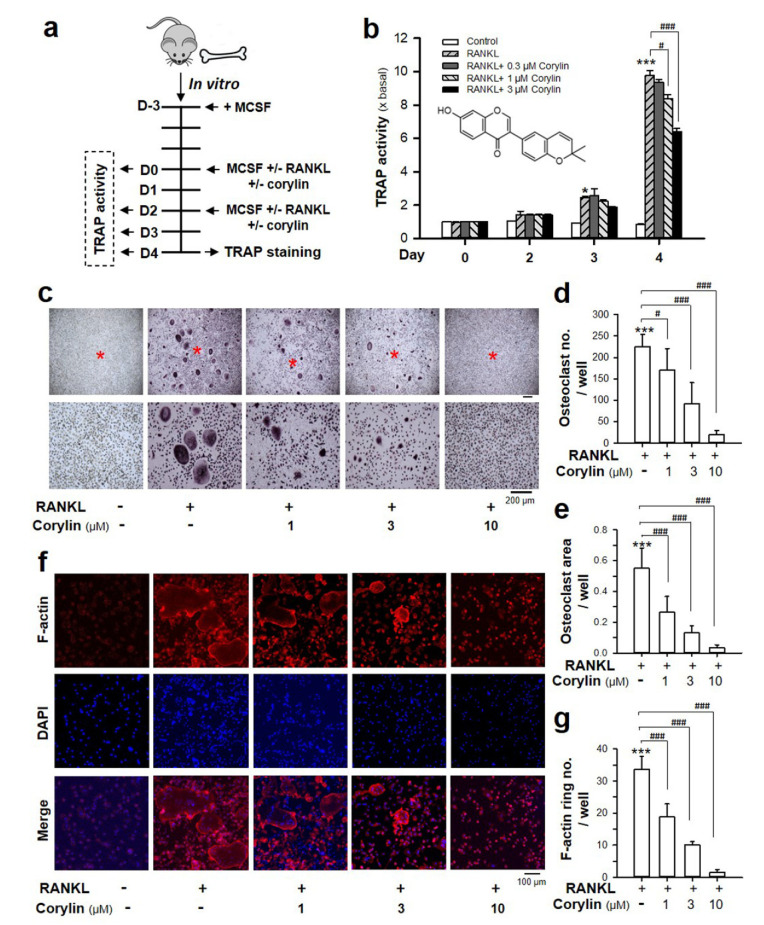
Corylin suppresses receptor activator of nuclear factor κB ligand (RANKL)-induced osteoclastogenesis. (**a**) Experimental scheme of in vitro osteoclast differentiation using bone marrow macrophages from C57BL/6 donors for tartrate-resistant acid phosphatase (TRAP) activity and the TRAP staining assay. Bone marrow monocytes were incubated with macrophage colony-stimulating factor (MCSF) (20 ng/mL) for the first 3 days to differentiate the cells, then incubated with MCSF (20 ng/mL) and RANKL (20 ng/mL) in the presence or absence of various concentrations of corylin for different days. (**b**) Corylin (1–10 µM) inhibited the activity of TRAP in a dose- and time-dependent manners. (**c**) The effect of corylin (1–10 µM) on RANKL-induced osteoclastogenesis at day 4 is indicated by TRAP staining. The lower panel shows higher magnification of the upper panel, and the red star indicated the magnification center. (**d**) The number of TRAP-positive, multi-nucleated (>3 nuclei) osteoclasts was counted. (**e**) The area of TRAP-positive, multi-nucleated (>3 nuclei) osteoclasts was measured. (**f**,**g**) Corylin inhibited F-actin ring formation of osteoclasts at day 4. Osteoclasts with an F-actin ring were counted. Representative images of three independent experiment are shown. The value is expressed as mean ± SD, *n* = 3–4. * *p* < 0.05, *** *p* < 0.001, compared with control; ^#^ *p* < 0.05, ^###^ *p* < 0.001, compared with RANKL-treated control.

The cytotoxicity of corylin, a known phytochemical, toward osteoclast precursor cultures was determined at 24 and 48 h. Corylin at concentrations ranging from 0.3 to 30.0 μM did not change the cell viability of bone marrow macrophages (Figure 1). To probe the effect of corylin in osteoclastogenesis, bone marrow macrophages were cultured in Modified Eagle’s medium (MEM)-α with MCSF and RANKL in the presence or absence of corylin (Figure 2a). The activity of TRAP, an enzyme actively expressed by osteoclasts, was increased in a time-dependent manner after the stimulation of RANKL in cultures (Figure 2b). This activity was significantly inhibited by corylin in a concentration-dependent manner after 4 days of treatment; however, TRAP activity was not affected by corylin during the early stages of osteoclastogenesis, i.e., from 1–3 days of treatment (Figure 2b). In a TRAP staining assay, bone marrow macrophages in the control (MCSF only) showed no change. In the treatment of the RANKL control (MCSF + RANKL), cultured bone marrow macrophages obviously differentiated into multi-nucleated TRAP-positive cells, an indicative marker of osteoclasts (Figure 2c). A decreasing number and size of TRAP-positive cells were revealed as responding to corylin treatment in a concentration-dependent manner (Figure 2c–e). These results show that corylin inhibited osteoclast formation in a time- and concentration-dependent manner.

Formation of an F-actin ring in osteoclasts is commonly used as an indicator of its resorptive activity. To determine the formation of an F-actin ring, bone marrow macrophages were seeded onto 24-well plates and cultured with or without serial concentrations of corylin for 4 days in the presence of MCSF and RANKL. A typical F-actin ring of multi-nucleated osteoclasts was identified in the differentiated osteoclast, i.e., RANKL control; whereas the treatment of osteoclasts with corylin resulted in a significant reduction of the F-actin ring, again in a concentration-dependent manner (Figure 2f–g). These data were consistent with morphologic retraction observed in the TRAP staining assay, which further supports that corylin could attenuate osteoclast formation with the interference of cytoskeletal formation during osteoclastogenesis.

### 2.2. Corylin Suppresses Phagocytosis of Pre-Osteoclasts

**Figure 3:**
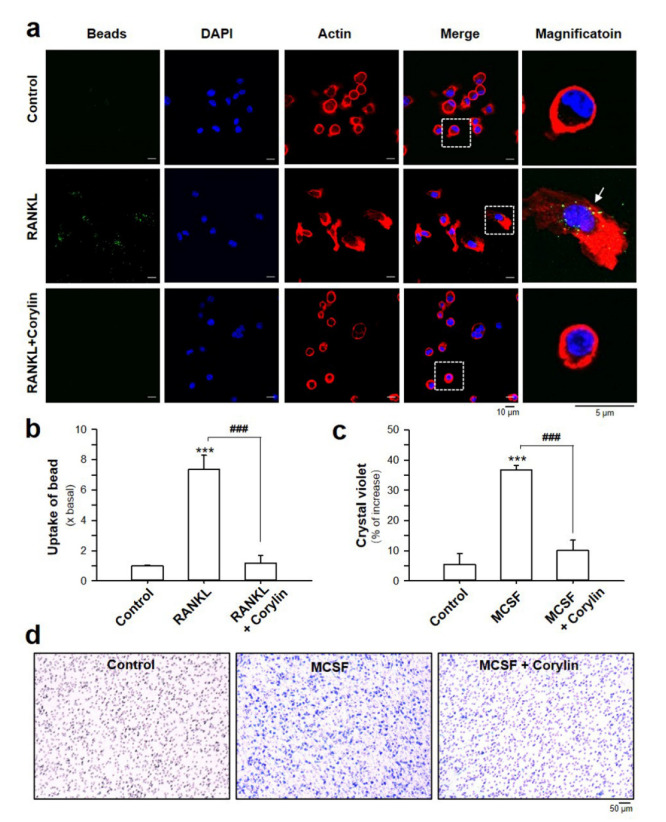
Corylin attenuates the phagocytosis and cell migration of pre-osteoclasts. (**a**) Bone marrow macrophages were prepared on glass coverslips, preincubated for 30 min with or without corylin (5 μM), and incubated for 40 min with fluorescent latex beads. Bone marrow macrophages were then fixed and stained with F-actin (red) and DAPI (blue) before observing by fluorescent microscopy. In the magnification picture, arrows indicate the bright green, fluorescent latex bead incorporated into the cell. (**b**) Phagocytic activity was expressed as the uptake of beads, calculated from the intensity of green fluorescent. (**c**) Quantification data of the effect of corylin on the cell migration from in vitro trans-well migration assay. (**d**) Representative images of crystal violet staining for migrated cells. Cells were seeded on the upper side of the trans-well membrane. Cell culture medium without fetal bovine serum (FBS) and conditioned medium (completed medium plus 30 ng/mL MCSF) were added to the lower chamber as the negative control and chemo-attractant, respectively. The value is expressed as mean ± SD, *n* = 3–4. *** *p* < 0.001, compared with control; ^###^ *p* < 0.001, compared with RANKL-treated control.

RANKL triggers the differentiation of bone marrow macrophages into pre-osteoclasts, and induces them to migrate and fuse with each other to form giant, multi-nucleated osteoclasts. Bone marrow macrophage and pre-osteoclasts are well known to act as phagocytes capable of ingesting latex beads [15]. This phagocytic capability of pre-osteoclasts allows the fusion of cells, which is important for the formation of mature and functional osteoclasts. To test whether the anti-osteoclastogenic activity of corylin was contributed by its potential to eliminate the phagocytosis of osteoclast precursor, the phagocytosis test, using latex beads and trans-well migration assay, was performed. As expected, the RANKL-stimulated bone marrow macrophages ingested more latex beads compared with the control (Figure 3a). However, the number of ingested latex beads was significantly decreased in the presence of corylin (Figure 3a,b), suggesting the inhibition of phagocytosis by corylin. In parallel, the results from the trans-well migration assay revealed that corylin significantly abrogated the MCSF-induced migration of pre-osteoclasts (Figure 3c,d).

### 2.3. Corylin Suppresses RANKL-Induced Gene Expression in Osteoclasts

To investigate the anti-osteoclastogenic properties of corylin, the expressions of transcription factors (NFATc1, c-Fos) and osteoclast-specific markers (TRAP, CTSK, calcitonin receptor (CTR), dendritic cell-specific transmembrane protein (DC-STAMP), matrix metalloproteinase-9 (MMP-9), and Atp6v0d2) in corylin-treated cultures were examined by RT-PCR. The mRNA expressions of RANKL-induced osteoclastic genes were inhibited by corylin in a time-dependent manner (Figure S1a). In addition, corylin suppressed the mRNA expression of RANKL-induced osteoclastic genes in a concentration-dependent manner (Figure S1b). The transcription factor NFATc1 was suppressed significantly after 4 days of corylin treatment (Figure S1b), and was nearly decreased by over half in the presence of corylin at 5 days of treatment (Figure S1a). Collectively, these data support the notion that corylin inhibits osteoclastogenesis via suppressing related gene expressions during the differentiation of bone marrow macrophages into osteoclasts.

### 2.4. Transcriptomic Analysis of Corylin-Treated Osteoclasts

**Figure 4:**
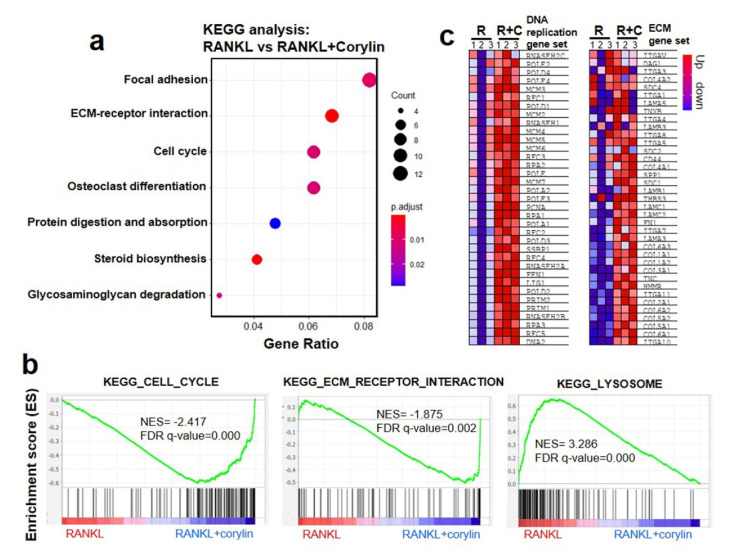
Transcriptomics of corylin-treated pre-osteoclasts. (**a**) Kyoto Encyclopedia of Genes and Genomes (KEGG) analysis of enriched signaling pathways in bone marrow macrophages treated with corylin for 4 days. The gene ratio is the ratio of DEG numbers to all gene numbers in a specific pathway. *P*-adjusted value is the corrected *p* value, ranging from 0 to 1. A greater rich factor and less *P*-adjusted value means a greater impact on the signaling pathway. (**b**) Gene set enrichment analysis (GSEA) enrichment plots using the KEGG gene set of an enriched “cell cycle” (normalized enrichment score (NES) = 2.417, false discovery rate (FDR) *q*-value < 0.000) and “ECM–receptor interaction” (NES = 1.875, FDR *q*-value < 0.002) in the RANKL group compared with RANKL + corylin group; meanwhile, the gene set related to lysosomes was enriched in RANKL (NES = 3.286, FDR *q*-value < 0.002) and down-regulated in the presence of corylin. Enrichment plots reveal the profile of the running enrichment score and positions of GeneSet members on the rank ordered list. (**c**) Heat map of the top features for each phenotype in gene expression. GSEA collapsed to gene symbols, representing a clustering of mRNA expression levels in cells treated with RANKL (R) or RANKL + corylin (R+C). Each column represents the indicated sample; each row indicates one gene. Expression values are represented as colors, where the range of colors (red, pink, light blue, dark blue) shows the range of expression values (high, moderate, low, lowest, respectively).

To explore the molecular details the underlying inhibition of corylin on RANKL-induced osteoclastogenesis, the transcriptome of RANKL-induced osteoclasts under corylin treatment was employed using RNA-seq. An osteoclast precursor was obtained and cultured as described in Section 4.4 and Figure 2a. Osteoclast precursors (cells cultured on day 0 in Figure 2a) were treated with corylin (5 μM) or the vehicle for 4 days. Kyoto Encyclopedia of Genes and Genomes (KEGG) analysis showed that corylin affected RANKL-induced osteoclastogenesis, mainly in osteoclastic differentiation, focal adhesion, extracellular matrix (ECM)–receptor interaction, and cell cycle (Figure 4a). Furthermore, gene set enrichment analysis (GSEA) using the KEGG gene set confirmed that genes related to the cell cycle (normalized enrichment score (NES) = 2.417; false discovery rate (FDR) *q*-value < 0.000) and ECM–receptor interaction (NES = 1.875, FDR *q*-value < 0.002) were enriched in the corylin-treated group compared to that with RANKL alone (Figure 4b,c); the genes related to osteoclast functions, e.g., the lysosome, were enriched in the RANKL group (NES = 3.286, FDR *q*-value < 0.002) and down-regulated in the corylin group, suggesting inhibitory activity of corylin in osteoclastic differentiation.

**Figure 5:**
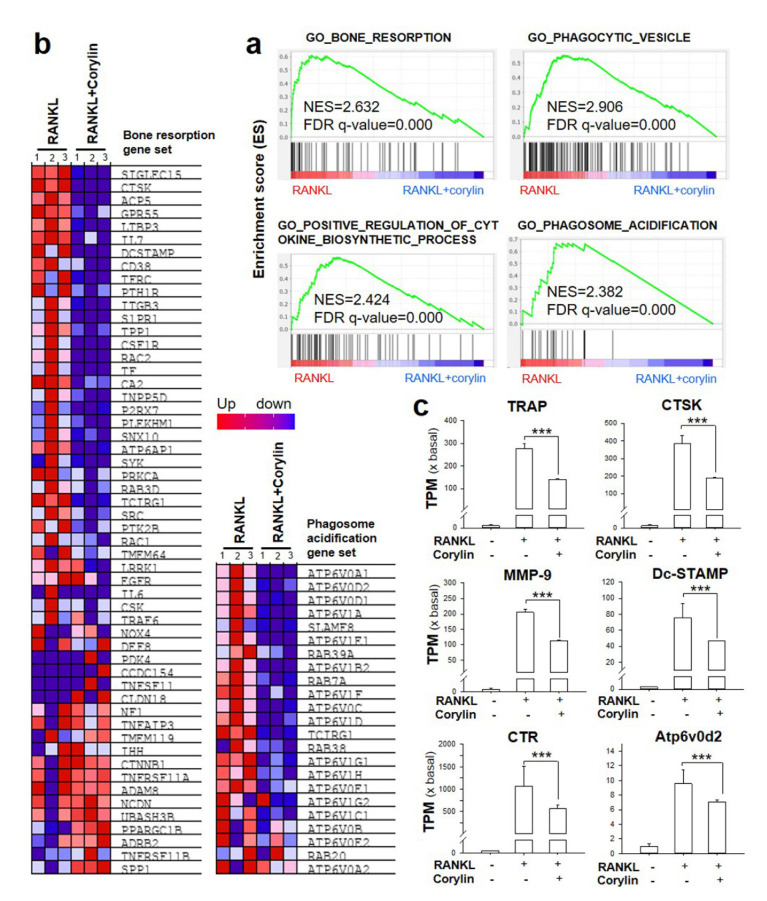
The gene set de-enriched by corylin in transcriptomics. (**a**) Transcriptomics were done from cultures, as in Figure 4. GSEA enrichment plots using the gene ontology (GO) gene set enriched “bone resorption” (NES = 2.632, FDR *q*-value < 0.000), “phagocytic vesicle” (NES = 2.906, FDR *q*-value < 0.000), “phagosome acidification” (NES = 2.382, FDR *q*-value < 0.000), and “positive regulation of cytokine biosynthetic process” (NES = 2.424, FDR *q*-value < 0.000). Enrichment plots revealed the profile of the running ES score and positions of gene set members on the rank ordered list. (**b**) Heat map of the top features for each phenotype in gene expression GSEA collapsed to gene symbols, representing clustering of mRNA expression levels in cells treated with RANKL or RANKL in the presence of corylin for 4 days. Each column represents the indicated sample; each row indicates one gene. Expression values are represented as colors, where the range of colors (red, pink, light blue, dark blue) shows the range of expression values (high, moderate, low, lowest, respectively). (**c**) The expression levels of osteoclast markers (TRAP, cathepsin K (CTSK), CTR, dendritic cell-specific transmembrane protein (DC-STAMP), MMP−9, and Atp6v0d2) in bone marrow macrophages treated with 5 μM corylin + RANKL for 4 days. The value is expressed as fold to control (x basal; vehicle), in mean ± SD, *n* = 3. *** *p* < 0.001, compared with RANKL-treated control. TPM: transcripts per kilobase million.

Furthermore, GSEA using the gene ontology (GO) gene set revealed that “phagocytic vesicle” was indicated as the most significantly associated GO term with an NES of 2.906 (FDR *q*-value < 0.000) (Figure 5a). Additionally, “bone resorption” (NES = 2.632, FDR *q*-value < 0.000), “phagosome acidification” (NES = 2.382, FDR *q*-value < 0.000), and “positive regulation of cytokine biosynthetic process” (NES = 2.424, FDR *q*-value < 0.000) were obviously enriched in the RANKL control group and down-regulated in the presence of corylin. (Figure 5a,b). Moreover, RNA-seq also showed that the expressions of several key makers (TRAP, CTSK, CTR, DC-STAMP, MMP-9, and Atp6v0d2) of osteoclasts were significantly enhanced in RANKL-treated bone marrow macrophages, which was diminished by corylin significantly (Figure 5c). These data were consistent with the results obtained by RT-PCR, which further confirmed that corylin suppressed RANKL-induced gene expression in osteoclasts. In summary, the results consolidate the inhibitory effect of corylin on RANKL-induced osteoclastogenesis.

### 2.5. Corylin Suppresses NF-κB Signaling Pathway in Osteoclastogenesis

**Figure 6:**
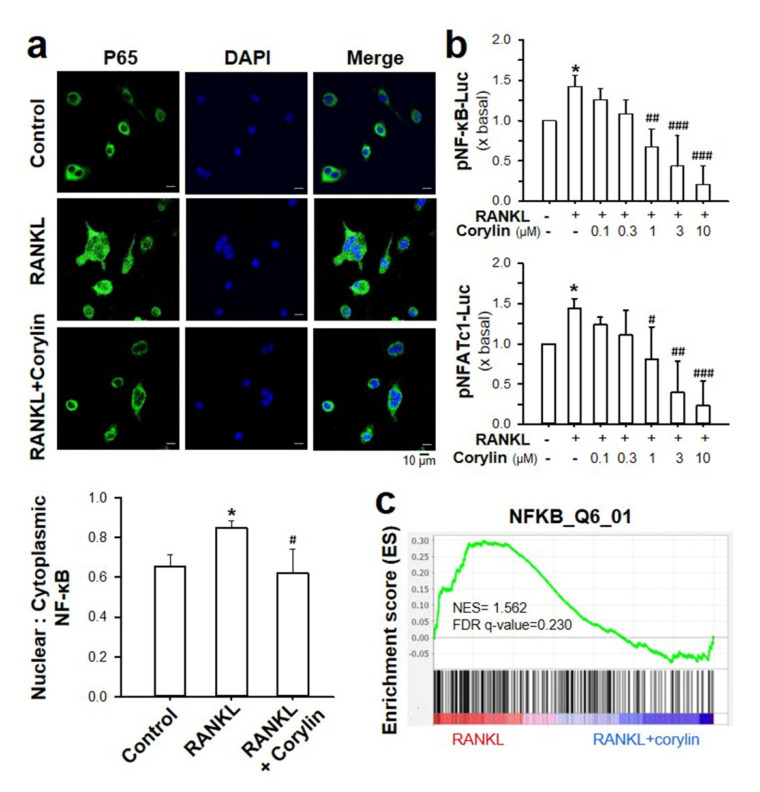
(**a**) Bone marrow macrophages were pre-treated with corylin for 4 h, followed by stimulation with 50 ng/mL RANKL for 15 min. Nuclear factor kappa-light-chain-enhancer of activated B cells (NF-κB) p65 localization was visualized by performing immunofluorescence staining. Nuclei were counterstained with DAPI. NF-κB p65 (green) and nuclei (blue) were examined under a fluorescence microscope. Quantification of the nuclear/cytoplasmic ratio of NF-κB p65 staining is shown in the lower panel. (**b**) Corylin regulates the transcription of NF-κB and nuclear factor of activated T cells c1 (NFATc1). Cultured RAW 264.7 cells were transfected with an NF-κB or NFATc1 promoter tagged with luciferase gene (pNF-κB -Luc and pNFATc1-Luc) before the application of RNAKL (50 ng/mL) and corylin (0.1–10.0 μM). pRL-TK vector was used as a control reporter. After 24 h incubation, cultures were collected for the luciferase assay. The results are presented as a fold of basal-normalized data arbitrarily set as 1, in mean ± SD, *n* = 3. * *p* < 0.05, compared with vehicle control; ^#^ *p* < 0.05, ^##^ *p* < 0.01, ^###^ *p* < 0.001, compared with RANKL-treated control. (**c**) GSEA enrichment plots using a transcription gene set enriched “NF-κB_Q6_01” (NES = 1.562, FDR *q*-value = 0.230). Enrichment plots reveal the profile of the running ES score and positions of gene set members on the rank ordered list.

RANKL-induced NF-κB activation is crucial in regulating osteoclastogenesis. To determine the signaling affected by corylin, immunofluorescence staining and a luciferase reporter assay were carried out. In the treatment of corylin, immunofluorescence staining showed an inhibition of nuclear translocation of NF-κB p65 by corylin (Figure 6a). In addition, the luciferase activities of pNF-kB and pNFATc1-Luc (NF-κB or NFATc1 promoter tagged with luciferase gene) in RANKL-treated cultures were markedly suppressed by corylin (0.1–10.0 μM), in a concentration-dependent manner (Figure 6b). Furthermore, GSEA using the transcriptional gene set revealed that the NF-κB gene set was obviously enriched in the RANKL control group and down-regulated in the corylin group (Figure 6c). Collectively, these data suggested that corylin affected NF-κB p65 nuclear translocation and NFATc1 expression during osteoclastogenesis.

### 2.6. Corylin Does Not Induce Osteoclast Apoptosis

**Figure 7:**
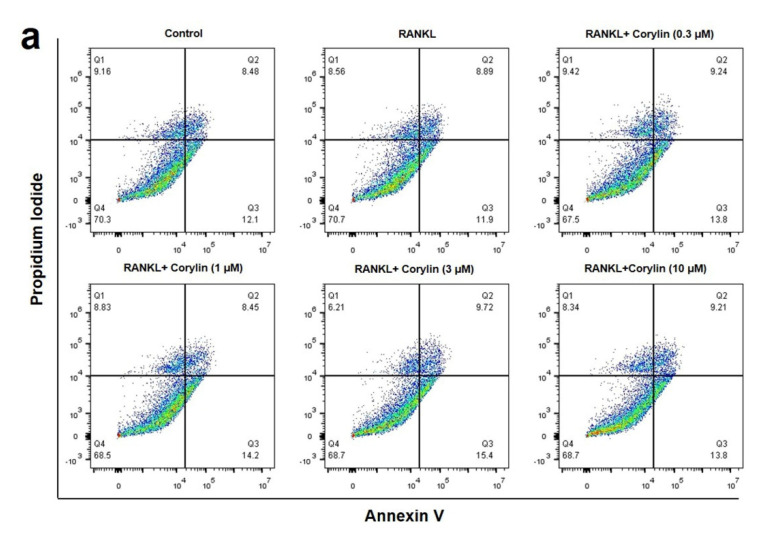

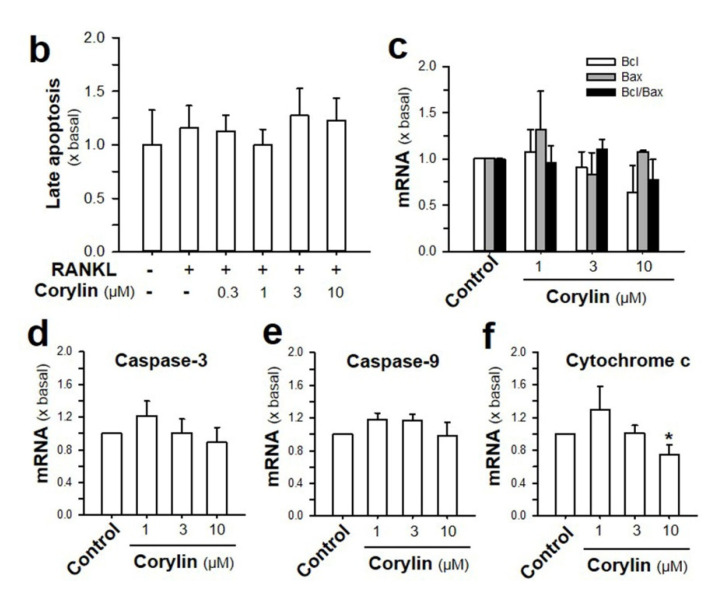
Corylin does not induce apoptosis in osteoclasts. (**a**) Bone marrow macrophages were cultured with 20 ng/mL RANKL in the presence or absence of corylin for 72 h. The cells were kept for 15 min in the dark for flow cytometry. (**b**) Quantification of late apoptotic cells form flow cytometry. Late apoptotic cells are propidium iodide (PI) and Annexin positive (PI/FITC +/+; Q2) (**c**–**f**) qRT-PCR analysis of mRNA expression of Bax, Bcl-2, caspase-3, caspase-9, and cytochrome c in bone marrow macrophages treated with corylin in the presence of RANKL (20 ng/mL) for 72 h. The value is expressed as fold to control (without corylin treatment), in mean ± SD, *n* = 4. * *p* < 0.05, compared with control.

The NF-κB signaling pathway has been shown to regulate cell apoptosis [16]. The question was raised whether corylin could have effect on osteoclast apoptosis. To answer this question, certain approaches, including flow cytometry and RT-PCR, were employed here. Corylin did not induce apoptosis of RANKL-treated bone marrow macrophages (Figure 7a,b). Neither the RANKL-treated control, nor corylin treatment in the presence of RANKL, showed a significant difference in the late apoptosis rate when compared with the control. Besides, the mRNA expressions of Bax, Bcl-2, caspase-3, caspase-9, and cytochrome c in the RANKL-stimulated pre-osteoclasts were not affect by corylin (Figure 7c–f). These results suggested that corylin exerted no effect on the apoptosis of the RANKL-stimulated osteoclast.

## 3. Discussion

Excessive formation or activity of osteoclasts is largely responsible for bone loss in skeletal disorders, including osteoporosis or rheumatoid arthritis [17]. The anti-resorptive therapeutics, e.g., bisphosphonates and RANKL inhibitors, have been approved to treat osteoporosis. However, it is worth exploring alternative drugs for osteoporosis, because the current drugs are associated with high cost and side effects. Osteoclasts are a key target in developing potential new drugs to treat osteoporosis. The present study shows a natural flavonoid—corylin, derived from the fruit of *P. corylifolia*—that could inhibit RANKL-induced osteoclastogenesis and gene expressions of osteoclast-specific markers via suppressing NF-κB signaling. Having robust effects in both osteoblasts and osteoclasts, our current finding suggests the further development of corylin as a potential drug against osteoporosis.

Osteoclastic differentiation and function are controlled primarily by MCSF and RANKL: both factors are expressed by osteoblast/stromal cell, as well as by signaling downstream from receptors in an osteoclast precursor [18,19]. MCSF induces the expression of receptor activator of nuclear factor kappa B (RANK), the receptor for RANKL, in osteoclast precursors [20]. Moreover, the binding of RANKL to RANK initiates the differentiation of osteoclast precursor into mature osteoclasts via activating the downstream signaling of NF-κB, which is followed by the sequential activation of transcription factors NF-κB, c-Fos, and NFATc1 [21,22]. Two lines of evidence, as revealed here, suggest that the inhibition of corylin in RANKL-induced osteoclastogenesis was via NF-κB signaling: (i) the reduction of NF-κB p65 nuclear translocation, and (ii) the suppression of NF-κB and NFATc1 transcription factors [23]. The inhibitory effect of corylin on RANKL-induced osteoclastogenesis via NF-κB signaling pathways subsequently raises our curiosity about the target of corylin. Soluble and membrane-anchored RANKL forms a homotrimer [24] that trimerizes RANK receptors [25], followed by recruiting TNF receptor-associated factor 6 (TRAF6) to the RANK receptor to induce downstream signaling. Therefore, we hypothesized that RANKL, RANK, or TRAF6 possibly could be the interacting target of corylin. However, our results from binding analysis and molecular docking did not support this notion.

NFATc1, a downstream effector of RANKL signaling and a master transcription factor regulating the later stage of osteoclastic differentiation, promotes the expression of genes crucial for osteoclast maturation and function through cooperation with c-Fos [26,27,28]. Here, we have shown that corylin exhibited anti-osteoclastogenic effects through the transcriptional response of NFATc1: the expression of NFATc1 subsequently regulates the transcription of target genes, e.g., TRAP, CTSK, CTR, DC-STAMP, MMP−9, and Atp6v0d2. Supporting bone resorption, TRAP, together with CTSK, is a prerequisite for bone resorption by osteoclasts. DC-STAMP and Atp6v0d2 are key molecules in mediating the fusion of pre-osteoclasts [29,30]. MMP−9, a type IV collagenase, is highly expressed in osteoclasts, and plays an important role in cell migration and degradation of the extracellular matrix [31]. The aforementioned results therefore support the notion of inhibitory actions of corylin on TRAP activity, F-actin ring formation, and osteoclast migration. In parallel, the RNA-Seq result shows that the transcripts related to bone resorption were up-regulated in RANKL-treated cells, but down-regulated in the presence of corylin. Moreover, corylin promoted cell cycle and ECM–receptor interaction, compared with cells treated with RANKL alone. This suggested that corylin might have an interaction with RANKL, because the function of RANKL is to induce the differentiation of bone marrow macrophages into osteoclasts. It is also reasonable to speculate that the influence of corylin on the cell cycle may be due to its action on NF-κB signaling, because NF-κB signaling has been reported to regulate the cell cycle [32].

Increasing apoptosis of osteoclasts could offer a useful target in treating bone diseases [33]. The process of apoptosis is initiated through two independent pathways, i.e., the death receptor and mitochondrial apoptosis pathways [33,34]. The latter is achieved by regulating the amounts of anti-apoptotic protein Bcl-2 and pro-apoptotic protein Bax upon cellular stimuli. The process regulates the release of cytochrome c into the cytosol from the mitochondria, which is indispensable for the formation of apoptosomes and caspase-9 activation during apoptosis [34]. Phytoestrogens, e.g., kaempferol and quercetin, have been shown to inhibit osteoclastic differentiation from RAW 264.7 cells by promoting cell apoptosis [35]. In this context, we are expecting that corylin, being a phytoestrogen, might induce apoptosis in RANKL-treated pre-osteoclasts. However, corylin robustly diminished the formation of multi-nucleated giant cells from bone marrow precursors, but did not induce cell death, as shown here. This result was further confirmed by the unresponsiveness of Bcl-2, Bax, caspase-3, and caspase-9 to corylin treatment. This may be accountable by the inhibition of NF-κB signaling pathways in osteoclasts by corylin, as NF-κB signaling pathways has been shown to regulate cell apoptosis [16]. Similar to corylin, apigenin, another common flavonoid, has no effect on the apoptosis of osteoclasts, but it does have profound anti-osteoclastogenic effects in RANKL-treated bone marrow macrophages [36].

Plant-derived phytochemicals exert diverse beneficial effects on human health by acting on different systems. A growing number of reports have demonstrated the benefits of these phytochemicals in the prevention and treatment of osteoporosis. Indeed, daidzein, rutin, and hesperidin have been shown not only to stimulate the formation of osteoblasts, but also inhibit the bone resorption of osteoclasts, which, as a result, is able to alleviate osteoporosis [37,38,39]. Kaempferol and quercetin have been preliminarily shown to inhibit the differentiation and activation of osteoclasts in vitro [40,41,42]; however, the osteogenic effects of these flavonoids have not been fully revealed. Here, we have identified that corylin possesses a robust effect on osteoclastogenesis. Comparing to other bone-beneficial phytochemicals, corylin has the following exciting advantages: (i) having dual effects of increasing bone formation and decreasing bone resorption; (ii) having anti-inflammatory, antioxidant, and estrogenic properties, making it a multi-target therapeutic drug candidate; and (iii) inhibiting osteoclast formation directly without influencing osteoclast apoptosis. There is a potential for corylin as an osteoclast inhibitor for bone disease, e.g., rheumatoid arthritis and post-menopausal osteoporosis.

## 4. Materials and Methods

### 4.1. Animals

Wild-type C57BL/6 mice (8-week-old, female) were supplied by the Animal and Plant Care Facility (APCF) at Hong Kong University of Science and Technology (HKUST). All experimental procedures were approved by the Committee on Research Practice and APCF at HKUST, and Department of Health, Hong Kong. Experiments involving animals are reported in compliance with the ARRIVE guidelines [43].

### 4.2. Materials

Cell culture media and supplements were purchased from Invitrogen (Carlsbad, CA, United States), except for those that are specifically indicated. Corylin was purchased from Chengdu Herbpurify (Chengdu, China) and had a purity of over 99%. The corylin was dissolved in dimethyl sulfoxide (DMSO) to give a stock solution of 100 mM. MCSF and RANKL were purchased from R&D Systems (Minneapolis, MN, United States). The leukocyte acid phosphatase kit and all chemicals, including 3-(4,5-dimethylthiazol-2-yl)-2,5-diphenyl tetrazolium bromide (MTT), 17β-estradiol (E2), tartrate solution, and acetate solution were purchased from Sigma-Aldrich (St. Louis, MO, United States). Fluorescent latex beads (0.1 µm) was purchased from Thermo Fisher Scientific (Waltham, MA, United States).

### 4.3. Cell Culture

RAW 264.7 murine macrophages (ATCC Cat# TIB-71, RRID: CVCL_0493) were cultured in Dulbecco’s modified Eagle medium (DMEM), supplemented with 10% heat-inactivated FBS and 1% penicillin–streptomycin. Cells were incubated at 37 °C in a water-saturated 5% CO_2_ incubator. Cells were dislodged from the plate with a scraper for sub-passages. The culture medium was replaced with osteoclast induction medium (complete cultured medium plus 50 ng/mL recombinant mouse RANKL) with or without the tested compound. The medium was replaced every 2 days until the formation of mature multinuclear osteoclasts was observed.

### 4.4. Differentiation of Osteoclasts from Bone Marrow Macrophages

Osteoclasts were generated by the methods previously described [44,45], with minor modifications. To generate primary osteoclasts, whole bone marrow was isolated from the long bones (tibia and femur) of 8-week-old mice, following the previously described method [46]. Then, bone marrow monocytes from the whole bone marrow were isolated by density gradient centrifugation with Ficoll-Paque (GE Healthcare Bio Sciences AB, Uppsala, Sweden) and cultured in bone marrow macrophage induction media (MEM-α complete medium supplemented with 20 ng/mL recombinant mouse MCSF) at 37 °C in a 5% CO_2_ incubator for 3 days. After that, the culture medium was replaced with osteoclast induction medium (complete cultured medium plus 20 ng/mL recombinant mouse MCSF and 20 ng/mL recombinant mouse RANKL), with or without the tested compound. The medium was replaced every 2 days until the formation of mature multinuclear osteoclasts was observed.

### 4.5. Cell Viability Assay

Cells were cultured in a 96-well plate, and MTT in 1× PBS at a final concentration of 0.5 mg/mL was applied onto cells for 3 h after drug treatments for 24 or 48 h. After the solution was removed, the purple precipitate inside the cells was re-suspended in DMSO followed by measurement at 570 nm absorbance using a spectrophotometer (Multiskan FC Microplate Photometer, Thermo Fisher Scientific, Waltham, MA, United States). The cell viability was calculated as the percentage of the absorbance value of the control (without drug treatment), and the value of the control was 100%.

### 4.6. Tartrate-Resistant Acid Phosphatase (TRAP) STAINING

The differentiated osteoclasts were fixed and stained for TRAP, an enzyme marker of osteoclasts, using a leukocyte acid phosphatase kit, according to the manufacturer’s instructions. Briefly, cells were stained with naphthol AS-BI phosphate, freshly diazotized fast gamer GBC, and tartrate solution for 1 h at 37 °C, followed by counter-staining with a hematoxylin solution. A TRAP-positive, multi-nucleated cell (>3 nuclei per cell) was counted as an osteoclast. The photos of stained osteoclasts were taken using a Nikon Ni-U upright fluorescence microscope equipped with MBF Stereo Investigator Software and a color camera. Image analysis was performed with ImageJ software (NIH Image, Bethesda, MD). The number of osteoclasts per well was calculated by averaging the counts from eight separated views.

### 4.7. TRAP Activity Assay

The cell lysates were extracted with passive lysis buffer (Polyplus transfection, New York, NY, United States) for 15 min at room temperature. TRAP activity was measured by mixing the cell lysate with a substrate mixture at 37 °C for 1 h and shielding it from light. Then, the reaction was stopped by adding 50 μL of 3 M NaOH into each well of the 96-well plate, and absorbance was measured at 405 nm. The substrate mixture consisted of a p-nitrophenyl phosphate (pNPP) substrate solution (610 mM pNPP in a buffer containing 0.1 M glycine (pH 10.4), 1 mM MgCl_2_, and 1 mM ZnCl_2_) and tartrate acid substrate buffer (89.3 mM of tartrate solution and 0.1 M of acetate solution in deionized water). The volume ratio of pNPP substrate solution to tartrate acid substrate buffer was 100:1. The tartrate acid substrate buffer was pre-heated to 37 °C before adding to the pNPP substrate solution.

### 4.8. F-Actin Ring Immunofluorescence Assay

After the formation of mature osteoclasts from bone marrow macrophages, the cells were fixed with 4% paraformaldehyde in PBS (pH 7.4) for 10 min, followed by PBS washes. Cultures were permeabilized by 0.1% Triton X-100 in PBS for 10 min and blocked by 5% bovine serum albumin (BSA) in PBS for 1 h at room temperature before rinsing cells three times with PBS. Next, cells were stained with rhodamine phalloidin (Thermo Fisher Scientific, Waltham, MA, United States) at room temperature, in the dark, for 10 min. After rinsing the cells three times in PBS for 5 min each time, the samples were dehydrated serially with 50%, 75%, and 100% ethanol, and mounted with ProLong Gold Antifade Reagent with DAPI (Cell Signaling Technology, Danvers, MA, United States). Images of osteoclastic F-actin rings were then examined by a Zeiss Laser Scanning Confocal Microscope (Carl Zeiss, Wetzlar, Germany).

### 4.9. Phagocytosis of Fluorescent Beads

Bone marrow macrophages were cultured on 13-mm coverslips in 24-well plates in presence of MCSF (20 ng/mL), with or without RANKL and different concentrations of corylin. Bone marrow macrophages were further maintained in serum-free MEM-α for 4 h. Then, fluorescent latex beads (1:500) were added to the cultures for 40 min. After being washed with PBS four times, cells were fixed and stained with rhodamine-conjugated phalloidin to visualize F-actin, as well as with DAPI to visualize nuclei. The fluorescent intensity was analyzed by NIH ImageJ software.

### 4.10. Cell Migration Assay

The bone marrow macrophages were plated at 40,000 cells/well on 5-μm pore polycarbonate membrane inserts in 6.5-mm Transwell membranes (Corning, Tewksbury, MA, United States). MCSF (30 ng/mL) plus 10% FBS was added to the bottom chamber as a chemotactic agent. Cells were incubated for 6 h in the presence of corylin on the bottom chamber. Cells not migrated were removed from the top side of Transwell membrane using a cotton swab. Upon fixation with 3.7% paraformaldehyde, cells on the bottom side of the Transwell membrane were stained with 0.05% crystal violet solution. Crystal violet stain was solubilized using 100% methanol, and optical density was quantified at 540 nm.

### 4.11. Real-Time Polymerase Chain Reaction (RT-PCR)

Bone marrow macrophages were seeded in six-well plates and cultured in complete MEM-α containing 20 ng/mL MCSF and 20 ng/mL RANKL. Following the RANKL-induced osteoclastogenesis, bone marrow macrophages were treated with different concentrations of corylin (0.5–5.0 μM) for 4 days or with 5 μM corylin for 0 to 5 days. The total RNA from cultured osteoclasts was isolated by RNAzol RT reagent (Molecular Research Center, Cincinnati, OH, United States), and RNA was reverse-transcribed by Moloney murine leukemia virus reverse transcriptase (Invitrogen), according to the manufacturer’s instructions. Template cDNAs were subjected to RT-PCR using specific primers for individual markers, as shown in Table 1. A housekeeping gene (β-actin) was used as an internal control. RT-PCR was performed in a LightCycler 480 (Roche Molecular Biochemical, Indianapolis, IA, United States) using KAPA SYBR FAST qPCR kits, in accordance with the manufacturer’s instruction. The 2^−ΔΔCt^ method was used to calculate relative expression levels. The specificity of amplification was confirmed by a melting curve.

### 4.12. Immunocytochemistry

To determine NF-κB p65 nuclear translocation, bone marrow macrophages, grown on coverslips, were treated with corylin for 4 h, followed by stimulation with 50 ng/mL RANKL for 15 min. After treatment, coverslips were washed twice in PBS, and cells were fixed with 4% paraformaldehyde in PBS for 10 min, followed by PBS washes. Cultures were permeabilized by 0.1% Triton X-100 in PBS for 10 min and blocked by 5% BSA in PBS for 1 h at room temperature. Cultures were incubated overnight with an anti-NF-κB p65 antibody (1:200) at 4 °C in a humidified chamber, followed by an Alexa 488-conjugated anti-rabbit secondary antibody for 2 h in the dark at room temperature. Samples were dehydrated serially with 50%, 75%, and 100% ethanol, and mounted with ProLong Gold Antifade Reagent with DAPI. The samples were then examined by a Zeiss Laser Scanning Confocal Microscope with a 63× oil immersion objective. Quantification of the nuclear/cytoplasmic ratio of NF-κB p65 staining was calculated according to a previous method [47].

### 4.13. DNA Transfection and Luciferase Reporter Assay

The NF-κB-luciferase plasmid (pNF-κB-Luc) was purchased from SwitchGear Genomics (Menlo Park, CA, United States), which contains a firefly luciferase reporter under the control of the full-length promoter. A plasmid expressing the firefly luciferase reporter driven by NFAT elements, pNFAT-Luc, was purchased from BD Biosciences Clontech (Palo Alto, CA, United States). A reporter vector, pRL-TK (Promega, Madison, WI, United States), containing the *Renilla* luciferase gene, was used as a reference control. The reporter construct pNF-κB-Luc or pNFAT-Luc was co-transfected with pRL-TK control plasmid into the RAW 264.7 cells, using jetPRIME kit Transfection Reagent (Polyplus, New York, NY, United States) for 6 h and 24 h, respectively. Then, cells were treated with RANKL (50 ng/mL) and corylin (0.1–10 μM) for another 24 h. Cell lysates were then extracted with passive lysis buffer and assayed for the firefly and *Renilla* luciferase activities with a dual luciferase reporter assay system using a GloMax 96 Microplate Luminometer (Promega). All values were presented as firefly luciferase activity normalized to *Renilla* luciferase activity.

### 4.14. Analysis of Osteoclast Apoptosis

The effects of corylin on osteoclast apoptosis was quantified using the Annexin V-FITC apoptosis detection kit (BD Biosciences, Franklin Lakes, NJ, United States). Bone marrow macrophages were treated with corylin (5 μM) for 72 h, and then washed twice with PBS and gently re-suspended in Annexin V binding buffer at a concentration of 100 × 10^4^ cells/mL. Osteoclasts (10 × 10^4^ cells, 100 µL) were added to a 5 mL flow tube, and 5 µL Annexin V-FITC and 5 µL propidium iodide (PI) were transferred. The cells were incubated for 15 min at room temperature (25 °C) in the dark and measured at 488 nm by flow cytometry (BD Biosciences, San Jose, CA, United States) within 1 h. The results were analyzed using Flow Jo v7.6 software (FlowJo LLC, Ashland, OR, United States).

### 4.15. RNA Sequencing (RNA-Seq) Analysis

The osteoclast precursor was obtained and cultured as described in Section 4.4 and Figure 2a. Osteoclast precursors (cells cultured on day 0 in Figure 2a) were treated with corylin (5 μM) or vehicle for 4 days. Then, the total RNA was extracted from cells using RNAzol RT. Library construction and sequencing were performed by BGI Tech Solutions Co., Limited (Hong Kong, China). The libraries were sequenced on a DNBseq platform and 100 bp paired-end reads were generated. The RNA-Seq data were transformed into a log2 scale, and gene ontology (GO) analysis, Kyoto Encyclopedia of Genes and Genomes (KEGG) analysis, and gene set enrichment analysis (GSEA) [48] were performed to identify the functions and associated enriched pathways of differentially expressed mRNAs.

### 4.16. Statistical Analysis

Data were analyzed using SPSS 22.0 (SPSS Inc., Chicago, Illinois, United States). Results were expressed as means ± SD of at least three to four independent determinations, each performed in triplicate. Means were compared by one-way analysis of variance, followed by a post hoc test (Fisher’s projected least significant difference), with statistical significance set at *p* < 0.05. Protein concentrations were measured using the Bio-Rad protein assay, based on protocol provided by the manufacturer.

## 5. Conclusions

In conclusion, corylin restrained the migration, fusion, and commitment of osteoclast precursors to osteoclastogenesis, without influencing cell apoptosis. The molecular mechanism was revealed by transcriptomic analysis, validating inhibitory targets, e.g., NF-κB essential modulators, for anti-osteoclastogenic properties of corylin. This study contributes to our understanding of the basic mechanism in regulating osteoclastic differentiation from bone marrow precursors, and provides insight into the function of natural flavonoids. Moreover, this study should be helpful in the discovery and development of natural products as effective osteoclastic inhibitors in treating bone diseases.

## Figures and Tables

**Table 1:** Primers used in the real-time PCR.

Genes	Forward (5′−3′)	Reverse (5′−3′)
NFATc1	CAG CTG CCG TCG CAC TCT GGT C	CCC GGC TGC CTT CCG TCT CAT A
c-Fos	CCA GTC AAG AGC ATC AGC AA	AAG TAG TGC AGC CCG GAG TA
TRAP	TCC TGG CTC AAA AAG CAG TT	ACA TAG CCC ACA CCG TTC TC
CTSK	CTT CCA ATA CGT GCA GCA GA	TCT TCA GGG CTT TCT CGT TC
CTR	TGC TGG CTG AGT GCA GAA ACC	GGC CTT CAC AGC CTT CAG GTA C
MMP−9	CAA AGA CCT GAA AAC CTC CAA	GGT ACA AGT ATG CCT CTG CCA
Atp6v0d2	AAG CCT TTG TTT GAC GCT GT	TTC GAT GCC TCT GTG AGA TG
DC-STAMP	CTT GCA ACC TAA GGG CAA AG	TCA ACA GCT CTG TCG TGA CC
Bcl-2	GTG GTG GAG GAA CTC TTC AG	GTT CCA CAA AGG CAT CCC AG
Bax	AGC AAA CTG GTG CTC AAG GC	CCA CAA AGA TGG TCA CTG TC
Caspase-3	CCT CAG AGA GAC ATT CAT GG	GCA GTA GTC GCC TCT GAA GA
Caspase-9	AGT TCC CGG GTG CTG TCT AT	GCC ATG GTC TTT CTG CTC AC
Cytochrome c	GAG GCA AGC ATA AGA CTG GA	TAC TCC ATC AGG GTA TCC TC
β-actin	AGC CAT GTA CGT AGC CAT CC	CTC TCA GCA GTG GTG GTG AA

## Data Availability

All the data generated or analyzed during this study are included in this article. The raw sequence data can be accessed in the NCBI, with accession number PRJNA702629.

## Supplementary Materials

The following are available online at https://www.mdpi.com/article/10.3390/ijms22073540/s1, Figure S1: Corylin suppresses RANKL-induced osteoclast-specific genes.
