# Supplementary figures and images for: Biological Evaluation and Transcriptomic Analysis of Corylin as an Inhibitor of Osteoclast Differentiation
Source: Int J Mol Sci. 2021 Mar 29;22(7):3540. doi: 10.3390/ijms22073540 (PMC8036378; doi:10.3390/ijms22073540)

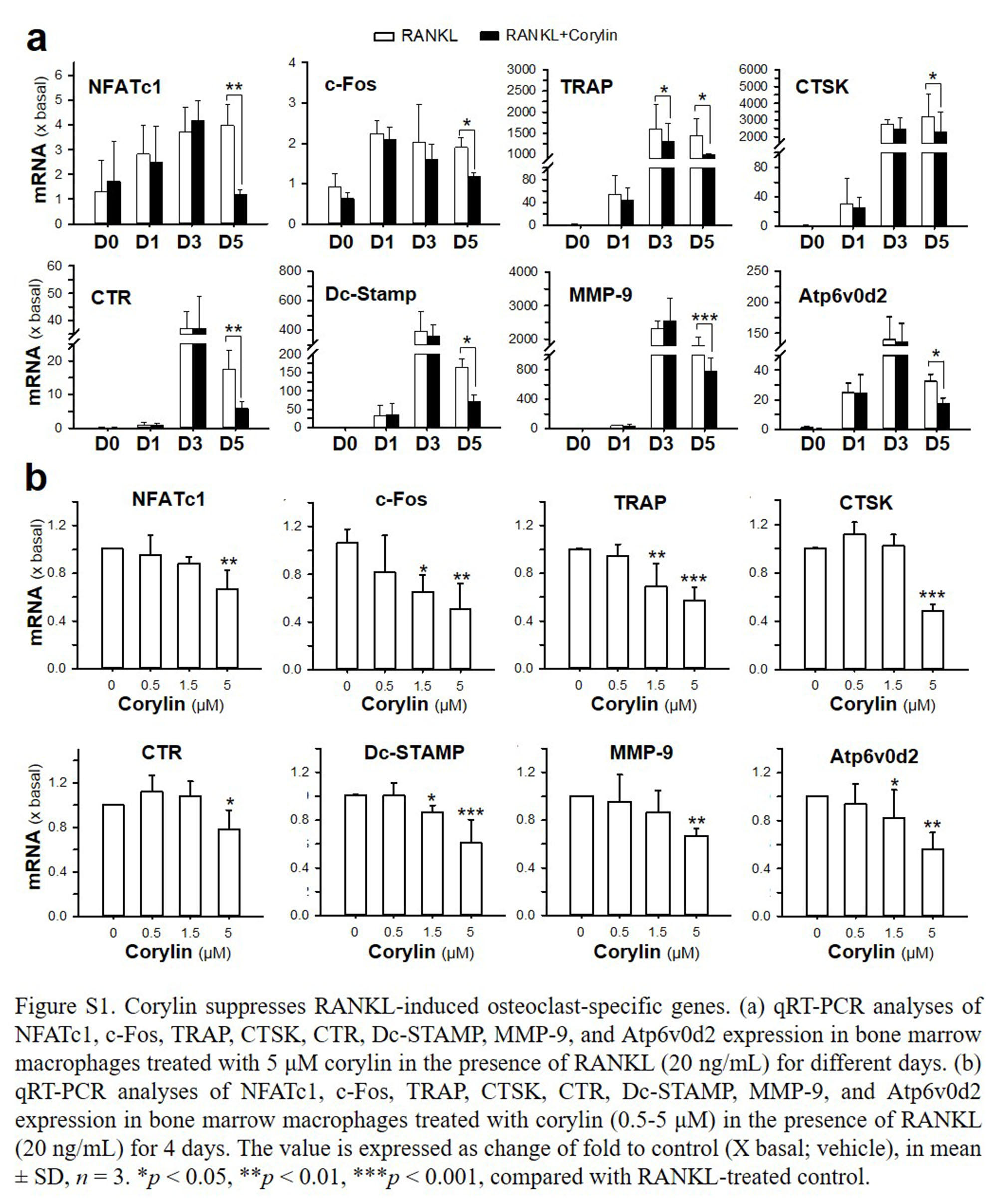

Supplement: Supplementary file 1 [file ijms-22-03540-s001.zip › ijms-1140179-supplementary.jpg]
